# Enteral feed obstructing its own way

**DOI:** 10.4103/0972-5229.74172

**Published:** 2010

**Authors:** Vikas Kesarwani, Dhaval R. Ghelani, Graham Reece

**Affiliations:** **From:** Department of Intensive Care Medicine, Blacktown Hospital, Blacktown Road, Blacktown NSW 2148, Australia

**Keywords:** Enteral feed solidification, esophageal obstruction, esophageal bezoar

## Abstract

Esophageal obstruction due to solidified enteral feeds is a rare but distressful complication in intensive care unit (ICU) patients. It has been suggested that gastroesophageal reflux, very low gastric pH, decreased pepsin and pancreatic enzyme secretions may be responsible for the solidification of casein containing enteral formulas. Recognition and avoidance of these factors will prevent such complication.

## Introduction

Early enteral feeding in critically ill patients in intensive care unit (ICU) is a fairly well-established approach. Amongst various complications associated with naso-gastric (NG) feeding, “diarrhea” and “tracheal aspiration” are the most common.[1,2] Esophageal obstruction due to the feed solidification is infrequent and has seldom been reported.

A patient in our ICU developed esophageal obstruction due to solidification of enteral feed, which led us to review our experience in the light of published literature. This case report and overview of pertinent literature intends to facilitate physicians to re-evaluate their approach to enterally fed patients, aiming to identify factors responsible for feed solidification.

## Case Report

A 71-year-old Caucasian man was admitted to our ICU with type 2 respiratory failure and severe hyponatremia. His past medical history included hypertension, gout, unilateral nephrectomy for renal tuberculosis and carotid endarterectomy.

The patient was intubated on the day of ICU admission for worsening respiratory failure and altered sensorium. A 14-French salem sump NG feeding tube was placed and NG tube tip position was confirmed on low chest radiograph to be in the stomach. Continuous infusion of polymeric, isotonic, fiber-mixed suspension (1 kcal/ml, Jevity^®^, Abbott Nutrition) was commenced and target feeding rate of 2000 kcal/day was achieved in 12 hours. The NG tube was flushed every 4-hourly with 30 ml of water. Medications administered through the NG tube during his ICU stay were metoprolol, lercanidipine, amlodepine, omeprazole, amiodarone, temazepam, folic Acid, coloxyl with senna and lactulose.

**Figure 1:**
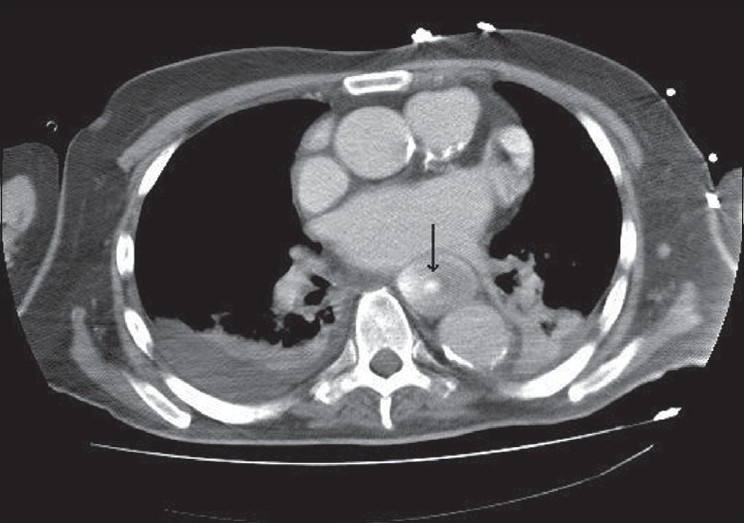
Chest CT scan showing intra-luminal space occupying lesion in the esophagus

The patient underwent tracheostomy on the 18^th^ ICU day after a failed extubation trial. Simultaneously, the NG tube was changed to 12-French polyurethane fine-bore tube. On the 47^th^ ICU day, a computerized tomography (CT) scan was done to delineate lung opacities seen on chest X-ray, and coincidentally, an intra-luminal space-occupying lesion in the esophagus was observed [Figure 1].

**Figure 2:**
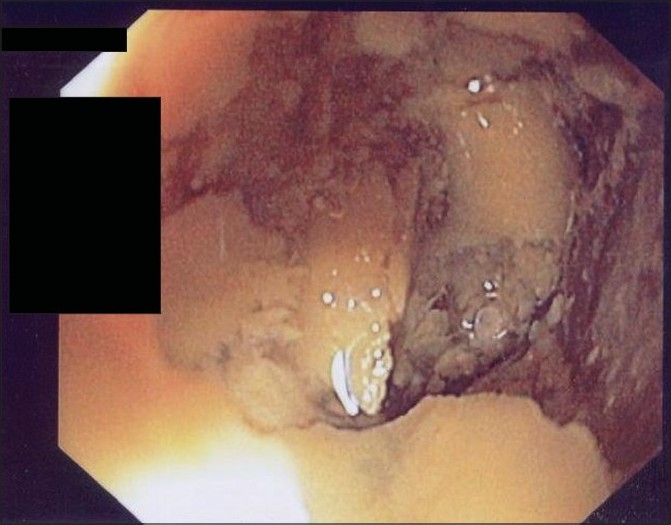
Endoscopic view of blocked esophagus with cheesy material

Concurrently, blockage of the NG tube necessitated its removal and a new NG tube could not be inserted beyond the hypopharynx. Fiber-optic esophagoscopy showed a white caseous substance with firm cheesy consistency completely obstructing the lumen of the distal esophagus [Figure 2]. This material could not be removed with the fiber-optic instrumentation, although at one time the esophagoscope went beyond the obstruction into the stomach with no evidence of any obstruction distally.

**Figure 3:**
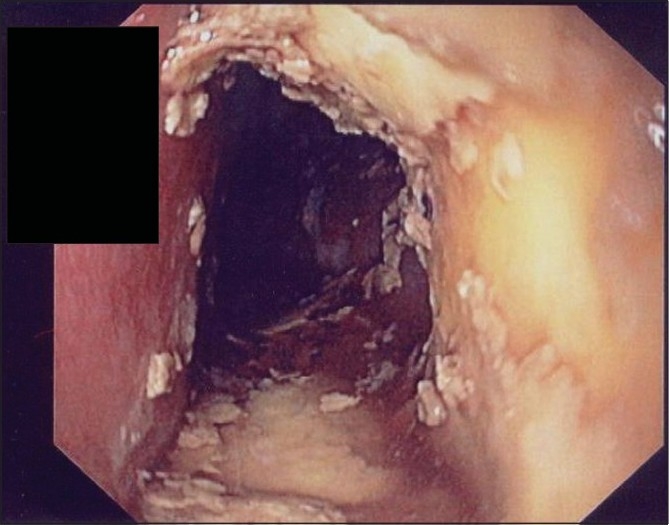
Endoscopic view of well-patent esophagus after scrapping of enteral feed

It took considerable time and three sittings over the next 3 days to scrap out the concretions from the lower 2/3^rd^of esophagus with biopsy forceps, suction and repeated washouts performed through a rigid esophagoscope. The obstructing material was not adherent to the esophageal mucosa [Figure 3]. The naked eye appearance of the removed material was the same as the solidified enteric feed.

Subsequently, a new NG tube was inserted and enteral feed recommenced. Over the next 2 weeks, the patient could be weaned from ventilator, was decannulated and discharged to the ward.

We could conclude that the esophageal obstruction had been caused by solidified enteric feed, as no other reason could account for such a clinical picture.

## Discussion

We searched Medline, Embase and Cumulative Index to Nursing and Allied Health Literature (CINAHL) from January 1980 to February 2009, using the following search terms: “nasogastric feed”, “enteral feed”, “oesophageal obstruction” and “oesophageal bezoar”. The literature appeared to be scattered and pointing toward various hypotheses postulated for the esophageal obstruction due to enteral feeding:

### 

#### Altered esophageal tone and motility coupled with gastroesophageal reflux

In mechanically ventilated patients, some degree of gastroesophageal reflux is unavoidable. Nasogastric tube can lead to loss of sphincter action of gastroesophageal junction with subsequent reflux of gastric acid and food contents from stomach.[3] Likewise, altered esophageal tone and motility will cause enteral feed stasis and precipitation.[4] By performing *in vitro* tests, Irgau *et al*.[5] demonstrated that stasis of the enteral feed formula and its fiber contents is not responsible for solidification by itself but is due to gastroesophageal acid reflux.

In radiological confirmation of the NG tube, the lower end of the tube is not always visualized. The chances of proximal hole being at or near the gastroesophageal junction are quite high, causing enteric feed delivery into the esophagus and gastric acid reflux precipitating it.

#### Enteral feeds containing casein protein precipitate in contact with acidic media of the stomach

Turner[6] and Myo[7] demonstrated that different compounds of enteral feed containing casein solidify in acidic medium (pH < 4.6) *in vitro*. However, solidification of feed did not occur with formulas not containing casein, viz., Clinifeed^®^ (Roussel Laboratories Ltd. Middlesex U.K.), which contains dried skimmed milk, and peptamen^®^ (Clintec Nutrition Ltd. Slough, U.K.), which contains peptides formed from hydrolysis of whey proteins, and these formulas remained liquid even at pH below 1 despite prolonged incubation at 37°C for 18 hours. Commercially available casein containing NG feeds are Osmolite^®^ (Abbott Nutrition), Ensure, Ensure Plus, Paediasure, Jevity, Pulmocare^®^ (Abbott Nutrition, Botany, NSW, Australia.), Fortison^®^ (Cow and Gate, U.K.).

#### Sucralfate as a cause of feed precipitation in esophagus

Many reported cases have implicated the use of sucralfate for the enteral feed precipitation.[4,8] Interestingly, laboratory findings by Rowbottom *et al*.[9] showed that increasing acidity (pH < 4) causes increased viscosity and precipitation of sucralfate and enteral feeding formulas, independently or when mixed together.

The aluminum content of sucralfate in acidic medium forms salts with dietary phosphates and leads to protein precipitation.[10]

#### Decreased pepsin and pancreatic enzyme secretions cause precipitation of gastric feed in acidic media

*In vitro* experiments conducted by Turner[6] and Myo[7] showed that acid-induced clotting of Osmolite^®^ (Abbott Nutrition, Botany, NSW, Australia) is inhibited by addition of pepsin and pancrex V (combination of pancreatic enzymes). This suggests that Osmolite^®^ (Abbott Nutrition) may solidify more readily when the action of pepsin and pancreatic enzymes is compromised. The patient described by Myo[7] had previous partial gastrectomy, suggesting reduced pepsin secretion, and low gastric pH may be responsible for the solidification of enteral feed.

The factors that led to the formation of esophageal bezoar in our patient were casein-rich tube feeding, gastric stasis and acid reflux.

We recommend the following strategies to prevent esophageal obstruction caused by solidification of enteric feed:

1. The proximal side-port and the distal tip of the NG tube should be visualized on a radiograph to be within the stomach before commencing and continuing enteral feeds.
2. Simple measures such as 30° head-up position will help prevent gastroesophageal reflux and gastric pooling.
3. Periodic flushing of the feeding tube will reduce the incidence of food stasis.
4. Avoid combining enteral formula and sucralfate.
5. In patients with peptic ulcer disease, significant gastroesophageal reflux and diseases with abnormal pepsin or pancreatic secretion; avoid casein-containing feeds and use adequate doses of acid lowering medications.
